# Empirical aspects of record linkage across multiple data sets using statistical linkage keys: the experience of the PIAC cohort study

**DOI:** 10.1186/1472-6963-10-41

**Published:** 2010-02-18

**Authors:** Rosemary Karmel, Phil Anderson, Diane Gibson, Ann Peut, Stephen Duckett, Yvonne Wells

**Affiliations:** 1 Faculty of Health, University of Canberra, Canberra, Australian Capital Territory, Australia; 2 Australian Institute of Health and Welfare, Canberra, Australian Capital Territory, Australia; 3 School of Population Health, University of Queensland, Brisbane, Queensland, Australia; 4 Lincoln Centre for Research on Ageing, Australian Institute for Primary Care, La Trobe University, Melbourne, Victoria, Australia

## Abstract

**Background:**

In Australia, many community service program data collections developed over the last decade, including several for aged care programs, contain a statistical linkage key (SLK) to enable derivation of client-level data. In addition, a common SLK is now used in many collections to facilitate the statistical examination of cross-program use. In 2005, the Pathways in Aged Care (PIAC) cohort study was funded to create a linked aged care database using the common SLK to enable analysis of pathways through aged care services.

Linkage using an SLK is commonly deterministic. The purpose of this paper is to describe an extended deterministic record linkage strategy for situations where there is a general person identifier (e.g. an SLK) and several additional variables suitable for data linkage. This approach can allow for variation in client information recorded on different databases.

**Methods:**

A stepwise deterministic record linkage algorithm was developed to link datasets using an SLK and several other variables. Three measures of likely match accuracy were used: the discriminating power of match key values, an estimated false match rate, and an estimated step-specific trade-off between true and false matches. The method was validated through examining link properties and clerical review of three samples of links.

**Results:**

The deterministic algorithm resulted in up to an 11% increase in links compared with simple deterministic matching using an SLK. The links identified are of high quality: validation samples showed that less than 0.5% of links were false positives, and very few matches were made using non-unique match information (0.01%). There was a high degree of consistency in the characteristics of linked events.

**Conclusions:**

The linkage strategy described in this paper has allowed the linking of multiple large aged care service datasets using a statistical linkage key while allowing for variation in its reporting. More widely, our deterministic algorithm, based on statistical properties of match keys, is a useful addition to the linker's toolkit. In particular, it may prove attractive when insufficient data are available for clerical review or follow-up, and the researcher has fewer options in relation to probabilistic linkage.

## Background

Since the 1980s the Australian Government has implemented numerous reforms to expand the focus of provision of aged care provision from residential care to include a wide range of community care services. Computerised person-level data are collected for administrative purposes for all residential care, but for only some community care programs. To fill this data gap, over the last 10 years several client-level national minimum datasets have been developed to provide regular information on government community service programs [1-3]. However, aged care programs generally have many distinct service providers, both government and non-government, and, for many programs, clients do not have a unique identifier within the program dataset that can be used to identify readily all of a person's program use.

To enable the derivation of client-level data, many of the community service program data collections contain a statistical linkage key (SLK) based on the concatenation of selected letters of name, date of birth and sex. The purpose of the SLK is to enable data linkage for statistical and research purposes while protecting client privacy, and not for client identification for administrative use or case management. In this context, "the process of linking client records does not need to be 100% accurate. Rather, statistical record linkage need only be sufficiently accurate to enable the drawing of statistically valid conclusions" [4].

In the Australian community services datasets the purpose of the SLK is then primarily to allow client-level analysis within particular government programs. However, when developing the datasets it was realised that the use of a common linkage key would greatly facilitate the statistical examination of cross-program use and care pathways. To allow for this possibility, a common SLK is now used in a number of data collections on government community services programs, including those relating to aged care and disability [1-3]. Linking the datasets is not routine, and requires approval from a properly constituted ethics committee (see [5]) and permission from all relevant data custodians.

Data linkage is a powerful tool both for identifying multiple appearances of individuals within a dataset and for integrating client information across datasets. As the information recorded for an individual may vary from dataset to dataset - due to either differences in reporting (e.g. in first name) or errors - a robust linkage process should allow for some discrepancy in reported characteristics. There are two main types of data linkage: probabilistic record linkage in which the linkage of records in two (or more) files is based on the probabilities of agreement and disagreement between a range of match variables, and deterministic record linkage in which the linkage of records is based on exact agreement of match variables.

Probabilistic matching allows for variation in reported characteristics by deriving a measure of similarity across variables used to identify matches, called the match weight. This is then used to decide whether a particular pair-wise comparison between records on two datasets is accepted (high weight) or rejected (low weight) as a match, or link [6,7]. Clerical review of possible record matches is often used to decide both the total weight above which record pairs are acceptable as a match and to determine whether matches with weights near this boundary should be considered to be valid [8-11]. Less commonly, a cut-off point for accepting matches is estimated using statistical models of probabilistic linkage, circumventing the need for clerical review [12]. In this approach detailed limited clerical follow-up may also be desirable to validate the process.

Simple deterministic linkage cannot allow for variation in reporting. However, deterministic algorithms can be constructed which can, and "[A]n intricate deterministic algorithm can be as successful - or more successful - than probabilistic algorithms in identifying valid links" [8].

Irrespective of method, when linking any pair of records four outcomes are possible: a true match (true positive), no match (true negative), a mis-match (false positive) and a missed match (false negative). In any linkage study, false negatives are caused by inconsistent reporting (or non-reporting) of data items across different datasets (i.e. data quality/consistency issues), while false positives are caused by different people, either rightly or wrongly, having common linkage data. False negatives are more likely to occur in simple deterministic matching than in probabilistic matching because client information may change depending on who provides the data and when the information is collected [13].

When linking using an SLK, additional data may be available that could assist record matching, with the auxiliary information providing a platform from which variation in the reported SLK information can be considered. The purpose of this paper is to describe a stepwise deterministic record linkage strategy for situations where there is a general person identifier (e.g. an SLK) and several additional variables suitable for data linkage. The auxiliary data may be available for nearly all clients or for a particular subset. This approach can allow for variation in client information across large databases in health and community care, and is demonstrated for the Pathways in Aged Care (PIAC) cohort study.

## Methods

### Current context: Pathways in Aged Care (PIAC) cohort study

Over a period people may access several community care programs and/or residential aged care. Coordination of these aged care services is important both to provide services cost-effectively and to provide the appropriate care for people at the appropriate time [14]. Between 2001-02 and 2005-06, four key programs in Australia accounted for around 85% of government expenditure on programs delivering community aged care (excluding assessment services) [15]. Datasets for these key community care programs, along with those for aged care assessments, residential aged care and deaths, are included in the PIAC study (see Table 1 for a brief description).

**Table 1:** Data in the PIAC project

Program	Description	Data source and years (client numbers)
Aged Care Assessment Program (ACAP)	Multi-disciplinary Aged Care Assessment Teams (ACATs) determine people's care needs and eligibility for RAC and packaged care (EACH and CACP), and make recommendations concerning the preferred long-term living arrangement.	National datasets: 2003-04 (105077) 2004-05 (141911)

Residential aged care (RAC)	RAC provides accommodation and care services to people who are no longer able to support themselves or be supported by others in their own homes, either permanently or for the short term (respite care). Care level required may be either 'low' or 'high'. Access requires approval via an ACAT assessment.	Administrative data: 1 July 2002 - 30 June 2006 (373183, including EACH)

Extended Aged Care at Home and Extended Aged Care at Home for people with Dementia (EACH)	Programs provide care at home that is equivalent to high-level residential care. Access requires approval via an ACAT assessment.	Administrative data: 1 July 2002 - 30 June 2006 (integrated with RAC data)

Community Aged Care Package program (CACP)	Program provides support services for older people with complex needs living at home who would otherwise be eligible for admission to 'low-level' residential care. CACPs provide a range of home-based services (excluding home nursing assistance and allied health services), with care being coordinated by the package provider. Access requires approval via an ACAT assessment.	Administrative data: 1 July 2002 - 30 June 2006 (80028)

Home and Community Care (HACC)	Program provides a large range of services (including allied health and home nursing services) to support people at home and to prevent premature or inappropriate admission to residential care. No ACAT assessment is required.	National datasets: 2002-03 (615642) 2003-04 (675446) 2004-05 (710781) 2005-06 (705261)

Veterans' Home Care (VHC)	Program provides a limited range of services to help veterans, war widows and widowers with low-level care needs to remain living in their own homes longer. Eligible veterans who need higher amounts of personal care than provided under VHC may be referred to other community care programs. No ACAT assessment is required.	Administrative data: 1 January 2001 - 28 February 2008 (164192)

National Death Index (NDI)	National register of deaths in Australia	Administrative data: 1 July 2003 - 30 December 2006 (415057 records)

*Note: *For details see [25-30]. See table 3.17 [30] for types of services provided by the aged care programs.

Client level data became available nationally for all the main aged care and assessment programs with the implementation in April 2003 of the person-level Aged Care Assessment Program (ACAP) national minimum dataset Version 2 [2]. However, the data for the various programs are held on different datasets so that analyses have continued primarily to be program-specific [16-19].

The aged care datasets do not share a common unique person identifier, and most do not contain full name data. Nevertheless, all either explicitly contain or have sufficient information to derive a common statistical linkage key termed SLK-581. This key, first proposed for the dataset for the Home and Community Care program [20], is the concatenation of five selected letters of name, eight digit date of birth and sex. Analyses have shown that SLK-581 distinguishes well between individuals in aged care datasets [20-22]. Consequently, the datasets can be linked using this SLK. In addition, depending on the datasets being matched, there are common data items - such as area of usual residence and event data - that could be used to aid the record linkage.

In 2005, a research team centred at the Australian Institute of Health and Welfare (AIHW) successfully applied for a National Health and Medical Research Council Strategic Award to undertake the PIAC cohort study. The purpose of this project is to create a linked dataset using data from the main aged care and assessment programs and to then undertake analyses of pathways in aged care over 24 months from the time of aged care assessment in 2003-04.

For the PIAC study, the cohort of interest is people who had a completed aged care assessment reported on the 2003-04 ACAP national dataset Version 2 (just over 105,000 people). The study required linking 10 datasets covering six aged care programs and deaths (Table 1). Deaths data were included to establish whether and when cohort members died within the study period. To be able to identify program use both before and after the reference year (2003-04), service use data from 2002-03 to 2005-06 were included. Aged care reassessments for 2004-05 were also identified.

Before data linkage was undertaken, ethics approval and permission to use the required data were obtained from all relevant bodies. In addition, to protect the privacy of individuals, all linkage was carried out within the AIHW using the Institute's data linkage protocol [23].

### Basic strategy

Data linkage for the PIAC cohort study was undertaken using multiple deterministic match passes in conjunction with an algorithm for identifying suitable match keys and the order in which they should be used. The deterministic match keys were based on (but not limited to) components of the common SLK. This approach was chosen because it does not rely on clerical review using name and/or address data - information which is not available on many of the datasets in the PIAC project.

**Figure 1:**
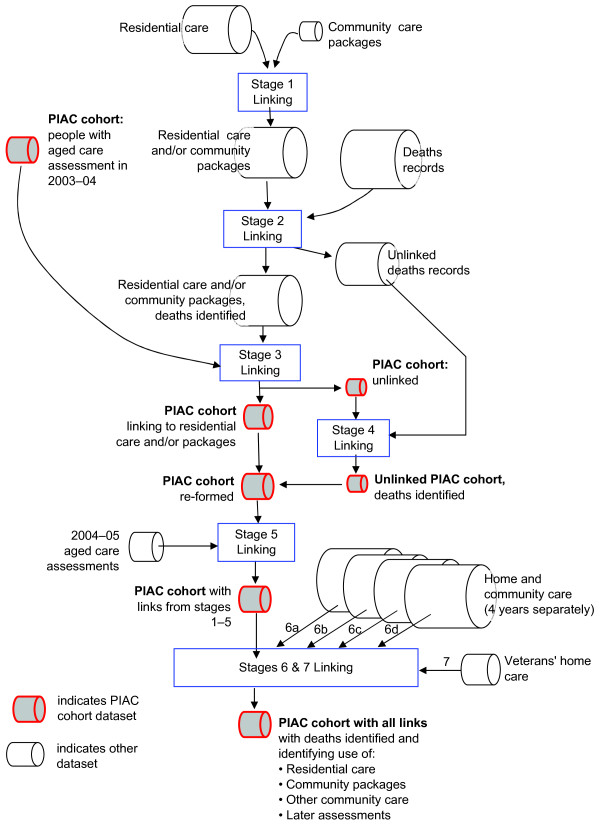
**Linkage stages for PIAC cohort study**.

To avoid unnecessary matching processes, a staged approach was employed which progressively linked the datasets two at a time (Figure 1). The order of linking the datasets was based on the availability of additional data for linkage and the quality of the linkage data. For PIAC, there were seven linkage stages in all, with match rates estimated to range from 3% to over 60%, depending on the stage (based on one-step deterministic matching using SLK-581, Table 2).

**Table 2:** Linkage stages in the PIAC project

Stage	Dataset 1	Dataset 2	Minimum match rate (from one-step deterministic linkage on SLK-581)		Final match rate (from stepwise deterministic linkage)	
			**% dataset 1**	**% dataset 2**	**% dataset 1**	**% dataset 2**

1	Residential care 2002-06^(a)^	Community care packages 2002-06^(a)^	9.4	44.0	10.2	47.7

2	Deaths July 03-Dec 06^(a)^	RCCP 2002-06^(a) ^(from stage 1)	32.6	36.9	36.2	41.0

**3**	**PIAC cohort (ACAP 2003-04)^(b)^**	**RCCP 2002-06^(a)^**	**64.4**	**16.3**	**72.6**	**18.4**

4	Deaths July 03-Dec 06 not linked to RCCP ^(a)^	ACAP 2003-04 not linked to RCCP^(b)^	3.0	31.4	3.3	34.7

5	PIAC cohort^(b)^	Aged care assessments 2004-05^(b)^	29.4	21.8	30.9	22.9

6a	PIAC cohort^(b)^	Home and Community Care 2002-03^(b)^	41.6	7.1	46.3	7.9

6b	PIAC cohort^(b)^	Home and Community Care 2003-04^(b)^	53.4	8.3	58.5	9.1

6c	PIAC cohort^(b)^	Home and Community Care 2004-05^(b)^	35.9	5.3	39.5	6.0

6d	PIAC cohort^(b)^	Home and Community Care 2005-06^(b)^	22.8	3.4	26.2	3.9

7	PIAC cohort^(b)^	Veterans' Home Care January 2001 - March 2008^(a)^	11.4	7.3	12.2	7.8

(a) Clients identified through administrative processes, and de-duplication.

(b) Clients identified by SLK-581 within broad region (1^st ^digit of postcode)

*Note: *For the datasets with administratively-derived person identifiers, data for all years in the study were linked at the same time. For data sets with clients identified by SLK-581 data were linked for each year separately to allow for variation over time in reported SLK-581.

The stepwise deterministic linkage strategy for each stage consisted of four phases. These are outlined below using stage 3 of the PIAC linkage to illustrate the processes (Figure 1). Stage 3 matches integrated residential care and community care packages (RCCP) data to the PIAC cohort as identified through the 2003-04 ACAP dataset. Simple deterministic matching using SLK-581 showed that at least 64% of the PIAC cohort would link to the RCCP dataset (Table 2).

#### Phase 1: Client identification within the two datasets

Individual clients are identified differently on the various datasets, depending on whether or not the dataset contains an administrative program client identifier. The RCCP dataset was derived in stage 1 from datasets with program client identifiers, and these were used to identify individuals. A small number of duplicate client records in the administrative datasets were identified and removed [21].

The ACAP dataset used to define the PIAC cohort does not contain either a unique program client identifier or name information, and so clients were defined using SLK-581 in conjunction with broad region of usual residence (first digit of postcode, which denotes Australian state or territory).

#### Phase 2: Identifying data for matching the specific dataset pair

The data to be used for linking are context-dependent. For stage 3, three additional variables were identified for matching in addition to SLK-581: postcode of residence, aged care assessment date and assessment team identifier.

The RCCP data can contain several postcodes for the same client over a year. When linking RCCP to the PIAC cohort two postcodes were allowed for: one community postcode and one relating to a residential care facility that the client had been in for permanent care during 2003-04.

#### Phase 3: Identification of keys to use in matching

Match keys to be used for linking were defined in terms of the SLK-581 (divided into five components) and additional available linkage data - postcode, aged care assessment date and assessment team identifier for stage 3. Many combinations of these eight variables could be used to define match keys (see, for example, Table 3 just using SLK-581 and region). To ensure that any employed match keys were based on combinations which both discriminated well between individuals and would not introduce too many false positives, a key was identified as suitable for matching using three criteria:

*Discriminating power:* 97.5% clients within each dataset had to have a unique value for the match key.

*Likelihood of introducing false matches:* The estimated theoretical false match rate for links established using the match key could be no more than 0.5%.

*Trade-off between additional true and additional false matches:* The estimated theoretical trade-off between additional true and additional false matches made with the key given matches already identified had to be at least 2 to 1.

**Table 3:** 128 keys based on components of SLK-581 and region

Key no.	Key description^(a)^	Key no.	Key description^(a)^	Key no.	Key description^(a)^
1	s3g2|dmyob|s|pc	44	s3g2|__yob|s|st	87	_g2|__yob|_|pc2
		
2	s3g2|dmyob|_|pc	45	_g2|dmyob|s|st	88	__|__yob|_|pc
		
3	s3g2|dm_ob|s|pc	46	s3_|dm_ob|_|pc2	89	s3g2|___ob|_|_
		
4	s3g2|dmyob|s|pc2	47	s3g2|__yob|_|st	90	_g2|dm_ob|_|_
		
5	s3_|dmyob|s|pc	48	_g2|dmyob|_|st	91	s3_|_yob|_|_
		
6	s3g2|dm_ob|_|pc	49	_g2|__yob|s|pc	92	s3_|___ob|s|pc2
		
7	s3g2|dmyob|_|pc2	50	s3_|dm_ob|s|st	93	__|dmyob|_|_
		
8	s3_|dmyob|_|pc	51	s3g2|__yob|s|_	94	__|dm_ob|s|pc2
		
9	s3g2|dmyob|s|st	52	_g2|dmyob|s|_	95	_g2|__yob|s|st
		
10	s3g2|dmyob|_|st	53	_g2|__yob|_|pc	96	s3_|___ob|_|pc2
		
11	s3g2|__yob|s|pc	54	s3_|dm_ob|_|st	97	__|dm_ob|_|pc2
		
12	_g2|dmyob|s|pc	55	s3g2|__yob|_|_	98	_g2|__yob|_|st
		
13	s3g2|dmyob|s|_	56	s3g2|___ob|s|pc2	99	s3_|___ob|s|st
		
14	s3g2|__yob|_|pc	57	s3_|___ob|s|pc	100	__|dm_ob|s|st
		
15	_g2|dmyob|_|pc	58	_g2|dmyob|_|_	101	_g2|__yob|s|_
		
16	s3g2|dmyob|_|_	59	_g2|dm_ob|s|pc2	102	s3_|___ob|_|st
		
17	s3g2|dm_ob|s|pc2	60	__|dm_ob|s|pc	103	__|dm_ob|_|st
		
18	s3_|dm_ob|s|pc	61	s3_|__yob|s|pc2	104	_g2|__yob|_|_
		
19	s3_|dmyob|s|pc2	62	__|dmyob|s|pc2	105	_g2|___ob|s|pc2
		
20	s3g2|dm_ob|_|pc2	63	s3_|dm_ob|s|_	106	__|___ob|s|pc
		
21	s3_|dm_ob|_|pc	64	s3g2|___ob|_|pc2	107	__|__yob|s|pc2
		
22	s3_|dmyob|_|pc2	65	s3_|___ob|_|pc	108	s3_|___ob|s|_
		
23	s3g2|dm_ob|s|st	66	_g2|dm_ob|_|pc2	109	__|dm__ob|s|_
		
24	s3_|dmyob|s|st	67	__|dm_ob|_|pc	110	_g2|___ob|_|pc2
		
25	s3g2|dm_ob|_|st	68	s3_|__yob|_|pc2	111	__|___ob|_|pc
		
26	s3g2|___ob|s|pc	69	__|dmyob|_|pc2	112	__|__yob|_|pc2
		
27	s3_|dmyob|_|st	70	s3_|dm_ob|_|_	113	s3_|_ob|_|_
		
28	_g2|dm_ob|s|pc	71	s3g2|___ob|s|st	114	__|dm_ob|_|_
		
29	s3g2|__yob|2|pc2	72	_g2|dm_ob|s|st	115	_g2|___ob|s|st
		
30	s3_|__yob|s|pc	73	s3_|__yob|s|st	116	__|__yob|s|st
		
31	_g2|dmyob|s|pc2	74	__|dmyob|s|st	117	_g2|___ob|_|st
		
32	__|dmyob|s|pc	75	s3g2|___ob|_|st	118	__|__yob|_|st
		
33	s3g2|dm_ob|s|_	76	_g2|dm_ob|_|st	119	_g2|___ob|s|_
		
34	s3g2|___ob|_|pc	77	s3_|__yob|_|st	120	__|__yob|s|_
		
35	s3_|dmyob|s|_	78	_g2|___ob|s|pc	121	_g2|___ob|_|_
		
36	_g2|dm_ob|_|pc	79	__|dmyob|_|st	122	__|__yob|_|_
		
37	s3g2|__yob|_|pc2	80	_g2|__yob|s|pc2	123	__|___ob|s|pc2
		
38	s3_|__yob|_|pc	81	__|__yob|s|pc	124	__|___ob|_|pc2
		
39	_g2|dmyob|_|pc2	82	s3g2|___ob|s|_	125	__|___ob|s|st
		
40	__|dmyob|_|pc	83	_g2|dm_ob|s|_	126	__|___ob|_|st
		
41	s3g2|dm_ob|_|_	84	s3_|__yob|s|_	127	__|___ob|s|_
		
42	s3_|dmyob|_|_	85	_g2|___ob|_|pc	128	__|___ob|_|_
		
43	s3_|dm_ob|s|pc2	86	__|dmyob|s|_		

(a) Key is a concatenation of data elements, indicated as follows:

• s3: the 2^nd^, 3^rd ^and 5^th ^letters of the family name substituting '2' for short names

• g2: the 2^nd ^and 3^rd ^letters of the given name substituting '2' for short names

• yob: year of birth

• dmob: day and month of birth

• s: sex

• pc: 4 digit postcode

• pc2: first two digits of 4 digit postcode

• st: state

• _: indicates that the component is not included in the match key.

Note: Order is from multiplying the number of the largest categories that account for roughly half of the clients within each key element (s3: 204, g2: 20, dmob: 182, yob: 16, s: 1, st: 2, pc2: 8, pc: 290). Key 13 is SLK-581.

The first of these criteria limits the testing of suitability to those keys that distinguish between individuals with reasonably high probability, while the second and third criteria ensure that an employed key adds few false matches given any matches which have been made in earlier passes. Appendix A provides details of the construction of measures used to implement these criteria, and presents estimates for stage 3.

Any combination of SLK-581 elements and additional match variables (i.e. potential match keys) that met all of the above criteria was used for matching the two datasets. Overall, 115 match keys were selected to match the PIAC cohort to the RCCP dataset, including 19 without ACAP assessment data.

#### Phase 4: Stepwise matching using selected match keys

Using the selected match keys, stepwise linkage was then carried out, with order of use determined by the discriminating power of the keys (going from high to low). Variation in match key elements identified through previous stages was also incorporated into match steps where relevant, resulting in the use of 215 versions of the 115 selected match keys. All links identified by the selected match keys were accepted as valid, with the exception of duplicate matches. In this case, a match was selected at random.

### Validation

Three methods were used to validate the stepwise deterministic linkage strategy. First, the quality of the data used to identify links was examined. Second, we analysed the distribution of identified links across the selected match keys and the consistency between client characteristics for linked pairs. Finally, clerical review of three random samples of links was undertaken to estimate the level of false matches.

## Results

For stage 3 of the PIAC linkage 72.6% of the PIAC cohort dataset linked to the RCCP dataset, corresponding to 18.4% of the RCCP dataset (Table 2). Comparing the achieved match rates with those based on simple matching on SLK-581 indicates that using extra information in the match keys (i.e. non SLK-581 data) allowed for variation in reported SLK-581 when matching. The quality of these links depends both on the quality of the data used to establish matches and on the ability of these data to distinguish between individuals. In the discussion below, the quality of the data used in the linkage is examined before analysing the results from the linkage process. The validity of established links is then discussed.

### Quality of match data

The presence of missing data reduces the likelihood of identifying true matches. The number of missed matches will also be relatively high if there are unreliable data on either of the datasets. For both datasets in stage 3 more than 97.8% of clients had complete SLK-581 data. However, the PIAC cohort dataset was more likely to have missing SLK-581 elements than RCCP data (2.1% versus 1.1%). In both datasets over 99.9% of client records with complete SLK-581 data had a unique SLK-581.

The availability of additional data (i.e. other than SLK-581) necessarily varies depending on the datasets being matched. For stage 3, 98.2% of the PIAC cohort dataset had valid postcode data. In the RCCP dataset, 99.0% of clients had at least one community postcode and 45.7% had a postcode relating to a residential care facility that the client had been in for permanent care during 2003-04.

Aged care assessment date and team data were available for all clients in the PIAC cohort dataset. On the other hand, many RCCP clients did not have an aged care assessment in 2003-04, and so these data were available for just 27% of all RCCP clients.

### Data linkage

Not all match keys identify links. Links between the PIAC cohort and RCCP data were made by 160 match keys out of the 215 used (including versions using previously identified SLK-581 and region variations).

The main purpose of a match key is to establish links. However, a close look at the list of keys used for stage 3 (see Table 4 and Table 5) suggests that many of the links would have been established using less discriminating - but still suitable - keys. Applying all selected keys in order has two advantages. First, using highly discriminating keys obviates the need to choose between records for clients with non-unique linkage information, thereby improving match quality. For stage 3, only 0.01% of matches were made between clients with non-unique match information for the identifying match key on either the PIAC cohort or RCCP datasets.

**Table 4:** Criteria for selecting match keys for PIAC stage 3 (examples for 60 keys)

Key no.	Linkage key	Joint. unique key rate (measure A)	^(a)^Est. number of links	Est. FMR (measure B)	^(b)^Comparison key	Marginal true: false (measure C)	^(c)^Est. 'worst case' FMR
1	s3g2|dmYOB|s|pc	99.999	55631	0.00	701	>1000	0.04

2	s3g2|dmYOB|_|pc	99.957	56120	0.00	702	>1000	0.09

3	s3g2|dm_ob|s|pc	99.878	57047	0.01	703	>1000	0.82

4	s3g2|dmYOB|s|pc2	99.993	63788	0.01	704	>1000	0.55

5	s3_|dmYOB|s|pc	99.896	56819	0.01	705	925.9	0.48

6	s3g2|dm_ob|_|pc	99.878	57547	0.02	706	578.7	1.63

7	s3g2|dmYOB|_|pc2	99.934	64338	0.02	707	592.1	1.09

8	s3_|dmYOB|_|pc	99.896	57326	0.03	708	466.2	0.95

9	s3g2|dmYOB|s|st	99.981	67206	0.04	709	317.7	1.93

10	s3g2|dmYOB|_|st	99.897	67781	0.08	710	159.5	3.82

11	s3g2|__YOB|s|pc	99.715	58484	0.12	711	103.9	15.40

12	_g2|dmYOB|s|pc	99.797	56031	0.14	712	88.2	3.17

13	s3g2|dmYOB|s|_	99.792	67743	0.17	713	80.7	5.74

14	s3g2|__YOB|_|pc	99.613	59012	0.23	714	51.9	30.52

15	_g2|dmYOB|_|pc	99.707	56541	0.27	715	44.0	6.28

16	s3g2|dmYOB|_|_	99.650	68327	0.29	716	44.9	10.23

17	s3g2|dm_ob|s|pc2	99.647	65447	0.34	717	36.9	10.16

18	s3_|dm_ob|s|pc	99.478	58319	0.41	718	28.9	8.84

19	s3_|dmYOB|s|pc2	99.583	65185	0.43	719	29.5	5.90

***20***	***s3g2|dm_ob|_|pc2***	***99.496***	***66024***	***0.67***	***720***	***18.1***	***20.14***

							

601	s3g2|dmYOB|s|pc	100.000	44977	0.00	. .	. .	0.00

602	s3g2|dmYOB|_|pc	99.998	45392	0.00	601	>1000	0.00

603	s3g2|dm_ob|s|pc	99.998	46105	0.00	601	>1000	0.01

604	s3g2|dmYOB|s|pc2	100.000	51170	0.00	601	>1000	0.00

605	s3_|dmYOB|s|pc	99.992	45855	0.00	601	>1000	0.00

606	s3g2|dm_ob|_|pc	99.998	46529	0.00	603	>1000	0.01

607	s3g2|dmYOB|_|pc2	99.998	51629	0.00	604	>1000	0.01

608	s3_|dmYOB|_|pc	99.992	46276	0.00	602	>1000	0.01

609	s3g2|dmYOB|s|st	100.000	53592	0.00	604	>1000	0.02

610	s3g2|dmYOB|_|st	99.998	54071	0.00	609	>1000	0.03

611	s3g2|__YOB|s|pc	99.976	47166	0.00	601	>1000	0.12

612	_g2|dmYOB|s|pc	99.978	45258	0.00	601	>1000	0.02

613	s3g2|dmYOB|s|_	100.000	53901	0.00	609	>1000	0.04

614	s3g2|__YOB|_|pc	99.962	47607	0.00	602	>1000	0.23

615	_g2|dmYOB|_|pc	99.976	45678	0.00	612	>1000	0.05

616	s3g2|dmYOB|_|_	99.998	54382	0.00	613	>1000	0.09

617	s3g2|dm_ob|s|pc2	99.994	52466	0.00	604	>1000	0.08

618	s3_|dm_ob|s|pc	99.986	47016	0.00	606	776.8	0.07

619	s3_|dmYOB|s|pc2	99.968	52178	0.00	604	>1000	0.05

620	s3g2|dm_ob|_|pc2	99.992	52936	0.00	617	772.5	0.16

							

701	s3g2|dmYOB|s|pc	100.000	49060	0.00	. .	. .	0.00

702	s3g2|dmYOB|_|pc	99.984	49502	0.00	701	>1000	0.00

703	s3g2|dm_ob|s|pc	99.957	50305	0.00	701	>1000	0.04

704	s3g2|dmYOB|s|pc2	99.996	55840	0.00	701	>1000	0.03

705	s3_|dmYOB|s|pc	99.952	50034	0.00	701	>1000	0.02

706	s3g2|dm_ob|_|pc	99.957	50757	0.00	703	>1000	0.07

707	s3g2|dmYOB|_|pc2	99.977	56333	0.00	704	>1000	0.05

708	s3_|dmYOB|_|pc	99.952	50486	0.00	702	>1000	0.04

709	s3g2|dmYOB|s|st	99.989	58515	0.00	704	>1000	0.09

710	s3g2|dmYOB|_|st	99.965	59029	0.00	709	>1000	0.18

711	s3g2|__YOB|s|pc	99.847	51479	0.00	701	>1000	0.70

712	_g2|dmYOB|s|pc	99.869	49369	0.00	701	152.5	0.14

713	s3g2|dmYOB|s|_	99.944	58836	0.01	709	144.2	0.26

714	s3g2|__YOB|_|pc	99.792	51951	0.01	702	679.1	1.39

715	_g2|dmYOB|_|pc	99.822	49816	0.01	712	220.5	0.29

716	s3g2|dmYOB|_|_	99.892	59352	0.01	713	174.0	0.52

717	s3g2|dm_ob|s|pc2	99.855	57266	0.01	704	251.2	0.46

718	s3_|dm_ob|s|pc	99.715	51314	0.01	706	91.6	0.40

719	s3_|dmYOB|s|pc2	99.803	56960	0.01	704	156.5	0.27

720	s3g2|dm_ob|_|pc2	99.796	57771	0.02	717	85.5	0.92

(a) Estimated number of links was derived from simple deterministic matching on the key (retaining only one occurrence of duplicates).

(b) Comparative linkage key is one which is slightly more detailed and includes all the match key elements of the current key. There is not a strict hierarchy for the linkage keys, so in some cases there may be more than one appropriate key for the comparison.

(c) 'Worst case' FMR is estimated assuming that the number of categories within a key element is equal to that implied by the most common category (s3: 72, g2: 11, dmob: 182, yob: 19, s: 2, st: 3, pc2: 11, pc: 156, aged care assessment date: 161, assessment team identifier: 25).

*Note: *See note to Table 3 for definition of keys; '600' series include assessment date; '700' series linkage keys include assessment team identifier. Table only includes keys that were expected to have fewer than four times as many people with non-unique match keys as SLK-581. This equates to key 20 if client region is the only additional match data, key 64 if aged care assessment date and region are included and key 46 if assessment team identifier and region are included. Keys in ***bold italics ***are those identified as not selected for use. Table showing all tested keys is available from the authors on request.

**Table 5:** Match results from stepwise matching for first 50 steps for PIAC stage 3

Step	Linkage key	Matches	Step	Linkage key	Matches
	
1	key_601	45031	26	key_610	15
	
2	key_601_2	904	27	key_610_2	.
	
3	key_601_3	1357	28	key_616	1
	
4	key_601_4	26	29	key_616_2	.
	
5	key_604	5151	30	key_617	113
	
6	key_604_2	99	31	key_617_2	2
	
7	key_604_3	.	32	key_617_3	6
	
8	key_604_4	146	33	key_617_4	.
	
9	key_609	2027	34	key_605	725
	
10	key_609_2	28	35	key_605_2	22
	
11	key_613	257	36	key_605_3	30
	
12	key_613_2	3	37	key_605_4	1
	
13	key_602	383	38	key_608	6
	
14	key_602_2	13	39	key_620	2
	
15	key_602_3	4	40	key_618	30
	
16	key_602_4	.	41	key_612	227
	
17	key_603	883	42	key_612_2	1
	
18	key_603_2	12	43	key_612_3	7
	
19	key_603_3	19	44	key_612_4	.
	
20	key_603_4	1	45	key_611	1697
	
21	key_606	8	46	key_611_2	13
	
22	key_607	36	47	key_611_3	52
	
23	key_607_2	.	48	key_611_4	1
	
24	key_607_3	3	49	key_615	3
	
25	key_607_4	.	50	key_623	53
**Summary of results**					

Matches with aged care assessment date (steps 1-105)					60780

Matches with assessment team identifier (steps 106-188)					7255

Other matches (steps 189-215)					8254

Total matches					76289

Legend for linkage key suffixes:

No suffix: linkage used preferred RCCP SLK-581 and preferred postcode

_2: linkage used alternative RCCP SLK-581 and preferred postcode

_3: linkage used preferred RCCP SLK-581 and alternative postcode

_4: linkage used alternative RCCP SLK-581 and alternative postcode

*Note: *See Table 3 for definition of keys. '600' series linkage keys use assessment date. Order of use is based on the joint unique key rate in Table 4. Table showing results for all selected keys is available from the authors on request.

Second, the range of elements included in keys can be used to show the relative strength of identified matches. Nearly 60% of stage 3 matches were made with the most detailed (i.e. first) match key. Also, while assessment event data were available for just over one-quarter of the RCCP clients, match keys incorporating these data identified 89% of the matches; however, only 1 in 50 matches needed this data to make the match. Furthermore, all but 0.4% of matches were made using keys for which the proportion of clients with a unique value was over 99.9%. These results indicate that the matches are highly reliable.

Using auxiliary information in the linkage increased the match rate by 11% for stage 3, with just over 90% of links between the PIAC cohort and RCCP datasets matching exactly on SLK-581. Just over 2% of matches were made using SLK-581 and region variations identified in stages 1 and 2. Also, 1.7% of links were for PIAC cohort members with incomplete SLK-581 information on the source ACAP dataset.

Data on region of residence was critical in identifying the additional matches, reflecting the high availability of this information, the strong discriminating power of residence in small regions and the relatively low availability of event data. Overall, 98% of the PIAC-RCCP matches could have been made by match keys which only used components of SLK-581 and region of residence.

### Linkage validation

Identified matches were consistent with other client information. Nearly 98% of the PIAC cohort whose assessment on the ACAP 2003-04 dataset was reported as being in residential care linked to the RCCP dataset. Also, there were few inconsistent links between the PIAC cohort, RCCP and deaths data: only 0.16% of the PIAC cohort had a link to a death record with a date of death before either an aged care assessment or program use identified by linking to RCCP data.

As permitted under project ethics approvals, link quality was explicitly investigated using the name information available on the deaths and RCCP datasets to review identified links clerically. Lack of full name on the PIAC cohort dataset prevented the RCCP to cohort linkage from being used for the comparisons.

Three samples of around 1000 RCCP-deaths links (from stage 2) were randomly selected and the full name, date of birth, sex, postcode and event data from the two data sources were manually compared to identify any false positives. Very few false matches were identified in these samples: 0, 1 and 5. This last was for the sample selected from among the 16128 links in which the SLK-581s of matched records were different. Because of the SLK differences, these links were considered to be more likely to be of low quality; however, even in this sample just 0.5% were identified as false positives.

## Discussion

Variation in linkage data when identifying matches deterministically was made possible by employing a set of three criteria for judging whether a combination of variables could be used to identify matches without resulting in too many false matches. A measure of a key's discriminating power was used to select a set of match keys to be investigated for use for linking two datasets. An estimated false match rate for each of these keys was then calculated and only match keys with an estimate under 0.5% were retained. Sometimes keys were further excluded on the basis that the estimated number of additional false matches was too high relative to the estimated number of additional true matches when allowing for matches made in earlier passes. All links identified by the selected match keys were accepted as valid, with the exception of the very small number of duplicate matches.

The occurrence of false negatives and false positives when matching between datasets is an issue faced by all studies using data linkage. Under-identification of matches is an expected limitation of linking deterministically using a single SLK, particularly when elements of the key may be missing or set to a default value when unknown. For the PIAC project, the occurrence of missed links was reduced through the use of methodical stepwise linkage using additional data items in combination with the SLK. This facilitated matches when the SLK had been reported inconsistently for individuals, including people with partially missing data in some datasets. In addition, by using the most discriminating match keys first, the algorithm aided correct matching in the small number of cases where different people had identical SLKs. These issues are particularly important for studies covering several years and multiple datasets, as people can report their name or date of birth differently on different occasions [13]. Data on the commonly available variable region of residence was critical in both situations.

The match key selection process and stepwise deterministic matching decreased the chances of making false matches by limiting match keys to those highly likely to lead to true matches, and by reducing the likelihood of duplicate matches. That this approach was successful is indicated by the high level of consistency observed in identified links and the very low false match rates seen in the clerical review of test samples.

The importance of allowing for variation in reported personal information (i.e. in the SLK) was demonstrated by the number of matches made using either identified SLK variations or reduced SLK data in conjunction with other information. Ten per cent of matches made in PIAC linkage stage 3 used a subset of the components of the SLK in combination with other variables, and 2.1% used SLK and region variations identified in earlier linkage stages. This shows that there is considerable variation in how people report their name and date of birth, with both the setting and reason for reporting personal information affecting how it is recorded on each occasion.

The amount of variation in the SLK allowed when linking various datasets depended on the availability of additional linkage information. However, additional data may not be available for all clients. The absence of additional linkage information means that less variation in the SLK can be allowed when identifying matches. Consequently, links for clients without data for additional match variables are more likely to be missed than those for other clients. However, in the PIAC linkage, additional information which was commonly unavailable for clients (i.e. event data) was necessary to identify only a small proportion of links (2% in stage 3), so that the overall effect on analyses is expected to be small.

## Conclusions

Simple deterministic matching is usually used to link datasets when matching using an SLK. However, such an approach leads to missing matches due to inconsistent reporting of SLK components. The linkage process for the PIAC cohort study has demonstrated that a stepwise deterministic matching algorithm with appropriate stopping rules can be used to allow for variation in reported personal information.

Three criteria were developed for judging whether a specific combination of variables could be used as a match key to identify matches without resulting in too many false matches. All three measures are readily derivable and provide a means of gauging the likelihood of making false matches and the trade-off between additional true and false matches when reducing the number of variables used to identify a match.

The importance of allowing for variation in reported personal information (i.e. in the SLK) was demonstrated in the current application by the number of matches made using reduced SLK data in conjunction with other information. The very low false match rates seen in sample comparisons using full name data demonstrated that the criteria employed to choose usable match keys restricted the number of false matches. This was confirmed by the high degree of consistency found both in the individual links and in the implied care pathways.

For the PIAC study, the linkage process described in this paper has allowed the compilation of a linked database that will enable a broad range of issues relating to Australian aged care services to be examined for the first time. In particular, it will provide information on the care actually accessed by clients following an assessment for service use and it will allow investigation into factors which influence entry to community or residential care. More widely, the deterministic matching algorithm used to link the various datasets in the PIAC cohort study relied on identifying suitable match keys using a set of criteria concerning their statistical properties. Similar criteria could be used in other linkage projects to allow for variation in client information across large databases in health and community care. Using a deterministic matching algorithm may be attractive in those circumstances when sufficient data are not available for the clerical review or follow-up that a researcher may like to include when using probabilistic linkage.

## Abbreviations

AIHW: Australian Institute of Health and Welfare; ACAP: Aged Care Assessment Program; FMR: False match rate; PIAC: Pathways in Aged Care; RAC: residential aged care; RCCP: residential care and community care packages; SLK: statistical linkage key; SLK-581: statistical linkage key for a person, being the concatenation of five letters of name (the 2^nd^, 3^rd ^and 5^th ^letters of the family name, and the 2^nd ^and 3^rd ^letters of the given name, substituting '2' for short names), date of birth (ddmmyyyy) and sex (1 for male or 2 for female)

## Competing interests

The authors declare that they have no competing interests.

## Authors' contributions

RK was the principal developer of the linkage strategy, undertook the data linkage and analysis and drafted the manuscript. PA provided statistical advice on developing the linkage strategy. DG and AP designed the study. SD provided advice on research design, particularly in relation to maximizing policy relevance. YW provided advice on the interpretation and use of the ACAP national dataset. All authors viewed and approved the manuscript for publication.

## Appendix A: Identification of match keys for stepwise deterministic matching in PIAC

Successive matches were made using different match keys at each pass, each match key being defined in terms of selected components of SLK-581 and additional available linkage data. Within a PIAC linkage stage, match keys were identified for linking by evaluating a large range of keys based on:

• three letters of surname (s3) in SLK-581 (always together)

• two letters of given name (g2) in SLK-581 (always together)

• day and month of birth (always together)

• year of birth

• sex

• region (using state, full four-digit postcode, or first two digits of postcode)

• additional event date for matching

• other additional data for matching.

The specific data used for linking at each stage were context-dependent.

There are 128 possible keys based on the presence or absence of the first six elements above, using the three regional groupings separately (Table 3).

Many of the keys listed in Table 3 are too broad to be considered for deterministic linking: on face value alone keys 100 to 128 would not be contemplated. To select keys to be considered for matching, only keys that were estimated to have fewer than four times as many people with non-unique match keys as SLK-581 were investigated. Keys 1 to 20 in Table 3 met this criterion. Using additional variables increases the number of theoretically possible distinct keys and hence the number of potential match keys that meet this criterion. The least specific keys investigated for use when aged care assessment date, assessment team identifier or date of death (in conjunction with region) were available were keys 64, 46 and 74, respectively, in Table 3.

In the absence of clerical review, the decision on whether a particular match key combination could be used for matching two particular datasets was based on three measures:

### Measure A

A measure of *discriminating power *(termed the joint unique key rate, and expressed as a percentage). This is the product of the unique key rates for the two datasets being linked, where the unique key rate is the proportion of records within a dataset that have a unique value for the key in question. A *discriminating power *of at least 95.0% was required for the key to be considered for matching, equating to a unique key rate of at least 97.5% within each dataset. This measure assumes that rare combinations of variables within a dataset are less likely than more common combinations to result in false matches between datasets.

### Measure B

An *estimated false match rate *(FMR) for links established using the match key. This had to be less than 0.5% for a key to be considered for the matching process.

### Measure C

*Estimated trade-off between additional true and additional false matches *for links established using the match key when compared with matches made by a slightly more precise key. The ratio of additional true to additional false matches had to be at least 2:1 for the key to be used for matching.

All three criteria had to be met by a potential match key for it to be included in the stepwise linking.

The above three measures were calculated prior to full linkage, with the latter two derived by applying the approximation methods outlined in Karmel and Gibson [24]. In the current context, when matching data for program 1 and program 2, FMR was approximated by

where

P is the size of the population (in 1000 s)

r is the usage rate of program 1 as indicated by its dataset (per 1000 people in population P)

α is the proportion of people using program 2 matching to those using program 1 when using a specific match key combination to match

β reflects the number of comparison cells specified by the components of the particular match key being used, allowing for uneven client spread across cells.

The linkage rate α for a specific match key was gauged by using only that key for simple deterministic matching. FMR was then derived (measure B), which also allowed estimation of the trade-off between true and false matches for a specific match key combination when compared with the results from a more exact key (measure C).

Using match keys that met the above criteria, stepwise linkage was then carried out, with order of use determined by the discriminating power of the keys (measure A, going from high to low). Missing information on common elements in a key could not lead to a match as missing data were set to different values in each dataset.

The key selection and stepwise matching processes are illustrated in Tables 4 and 5 for stage 3, which matches RCCP data to the PIAC cohort as identified through the 2003-04 ACAP dataset. For comparative purposes, an estimated 'worst case' false match rate is also presented in Table 4. Overall, 76289 matches were identified in stage 3: 60780 using keys incorporating assessment date, a further 7255 using keys with assessment team identifier, and 8254 using keys which did not include assessment data (Table 5). Under 600 matches (0.8%) were made using keys with an estimated 'worst case' FMR of more than 10% (just 15 matches with an estimated 'worst case' FMR of more than 20%).

Note that measure A is related to the 'global u probability' used in probabilistic matching to give a measure of how likely it is that two values will agree by chance [10]. The global u probability assumes that each value of a variable has the same probability and so is defined as

For the current application, within a dataset the global u for a specific match key is estimated by

where S is the size of the dataset (and assuming no more than 2 duplicates per key). For a key with a uniqueness rate of 97.5% within the dataset, then u ≈ 1/{0.9875*S}.

Note also that the proportions of matches made using keys incorporating a subset of elements of SLK-581 provide an indication of the likelihood of inconsistent reporting of name, sex and date of birth data; that is, they indicate the size of the relevant m probabilities used in probabilistic linkage, where the m probability is the probability that a data element is recorded identically for the same client on two occasions. Matches made using different levels of region data also provide a measure of consistency in reported region of residence.

## Pre-publication history

The pre-publication history for this paper can be accessed here:

http://www.biomedcentral.com/1472-6963/10/41/prepub

## References

[B1] Home and Community Care Program minimum data set, 2002-03 annual bulletin Australian Government Department of Health and Ageing http://www.health.gov.au/internet/main/publishing.nsf/Content/28B09A156480B595CA256F1900108686/$File/mds_sb0203.pdf

[B2] Aged Care Assessment Program National Data Repository Aged Care Assessment Program National Data Repository: minimum data set report, Annual Report 2003-04 2005 Melbourne: La Trobe University

[B3] Australian Institute of Health and Welfare (AIHW) Disability support services provided under the Commonwealth/State Disability Agreement, national data 1999. Canberra 2000

[B4] Ryan T Holmes B Gibson D A National Minimum Data Set for Home and Community Care 1999 Canberra: AIHW 76

[B5] National Health and Medical Research Council (NHMRC), Australian Research Council, Australian Vice-Chancellors' Committee National Statement on Ethical Conduct in Human Research 2007 Canberra: NHMRC

[B6] Fellegi IP Sunter AB A Theory for Record Linkage Journal of the American Statistical Association 1969 64 1183 1210 10.2307/2286061

[B7] Jaro M Probabilistic linkage of large public health data files Statistics in Medicine 1995 14 112 121 10.1002/sim.4780140510 7792443

[B8] Campbell KM Rule Your Data with The Link King^© ^(a SAS/AF^® ^application for record linkage and unduplication) SUGI 30: 2005; Paper 020-030 2005 Philadelphia: SAS http://www2.sas.com/proceedings/sugi30/020-30.pdf

[B9] Ascential Software Corporation WebSphere® QualityStage Version 8: User guide 2006 IBM

[B10] Data Integration Manual Statistics New Zealand http://www.stats.govt.nz/about_us/policies-and-guidelines/data-integration/further-technical-information.aspx

[B11] Herzog T Scheuren FJ Winkler WE Data quality and record linkage techniques 2007 New York: Springer

[B12] Meray N Reitsma JB Ravelli ACJ Bonsel GJ Probabilistic record linkage is a valid and transparent tool to combine databases without a patient identification number Journal of Clinical Epidemiology 2007 60 883 891 10.1016/j.jclinepi.2006.11.021 17689804

[B13] National Community Services Information Management Group Statistical data linkage in community services data collections: a report prepared by the Statistical Linkage Key Working Group 2004 Canberra: AIHW

[B14] Gray L Two year review of aged care reforms 2001 Canberra: DHAC

[B15] Hales C Peut A Mathur S, Gibson D Ageing and aged care Australia's welfare 2007 2007 Canberra: AIHW table 3.23.

[B16] AIHW Residential aged care in Australia 2004-05: a statistical overview 2006 Canberra: AIHW

[B17] AIHW Community Aged Care Packages in Australia 2004-05 2006 Canberra: AIHW

[B18] Aged Care Assessment Program National Data Repository Aged Care Assessment Program National Data Repository: minimum data set report, Annual Report 2004-05 2006 Melbourne: La Trobe University

[B19] Home and Community Care Program minimum data set, 2003-04 annual bulletin Australian Government Department of Health and Ageing http://www.health.gov.au/internet/main/publishing.nsf/Content/28B09A156480B595CA256F1900108686/$File/mds_annual_04.pdf

[B20] Ryan T Holmes B Gibson D A National Minimum Data Set for Home and Community Care 1999 Canberra: AIHW

[B21] Karmel R Data linkage protocols using a statistical linkage key 2005 Canberra: AIHW

[B22] Karmel R Transitions between aged care services 2005 Canberra: AIHW

[B23] Data linkage and protecting privacy: a protocol for linking between two or more data sets held within the Australian Institute of Health and Welfare Australia http://www.aihw.gov.au/dataonline/aihw_privacy_protection_protocols_data_linkage.pdf

[B24] Karmel R Gibson D Event-based record linkage in health and aged care services data: a methodological innovation BMC Health Services Research 2007 7 154 10.1186/1472-6963-7-154 17892601 PMC2254617

[B25] Gibson D Liu Z Choi C, Foard G, Gibson D, Madden R, Vaughan G Aged care Australia's Welfare 1993: services and assistance 1993 Canberra: AIHW 200 265

[B26] Gibson D Holmes B Liu Z Choi C, Gibson D, Goss J, Griffin J, Madden R, Madden R, Maples J, Moyle H, Wilson D Aged care Australia's Welfare 1999: services and assistance 1999 Canberra: AIHW 165 213

[B27] Gibson D Bowler E Angus P Braun P Mason F Madden R, Gibson D, Choi C, Maples J, Madden R Aged care Australia's Welfare 2001 2001 Canberra: AIHW 199 257

[B28] Karmel R Jenkins A Angus P Bowler E Braun P Gibson D, Madden R, Stuer A Ageing and aged care Australia's Welfare 2003 2003 Canberra: AIHW 275 329

[B29] Karmel R Peut A Bennett S Hogan R Gibson D, Abello R Ageing and aged care Australia's Welfare 2005 2005 Canberra: AIHW 134 201

[B30] Hales C Peut A Mathur S, Gibson D Ageing and Aged Care Australia's Welfare 2007 2007 Canberra: AIHW 77 152

